# Supplementary material for: RNA-Seq Reveals Extensive Transcriptional Response to Heat Stress in the Stony Coral Galaxea fascicularis
Source: Front Genet. 2018 Feb 13;9:37. doi: 10.3389/fgene.2018.00037 (PMC5816741; doi:10.3389/fgene.2018.00037)
Supplement: Supplementary Figure 1 — Changes of zooxanthellae densities in Galaxea fascicularis during heat stress treatment. [file Image1.PDF]

Supplementary Material

RNA-seq reveals extensive transcriptional response to heat stress in the stony coral *Galaxea fascicularis*

Jing Hou, Dingjia Su, Tao Xu, Ying Wu, Li Cheng, Jun Wang, Zhi Zhou*, Yan Wang*

*** Correspondence:**

Yan Wang, Zhi Zhou

ywang@hainu.edu.cn, zhouzhi@hainu.edu.cn

Supplementary Figures:

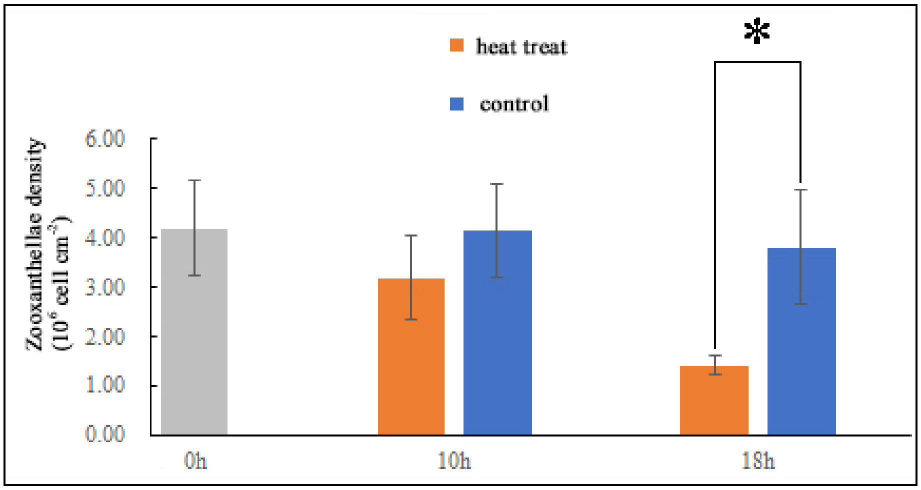

Supplementary Figure S1. Changes of zooxanthellae densities in *Galaxea fascicularis* during heat stress treatment.

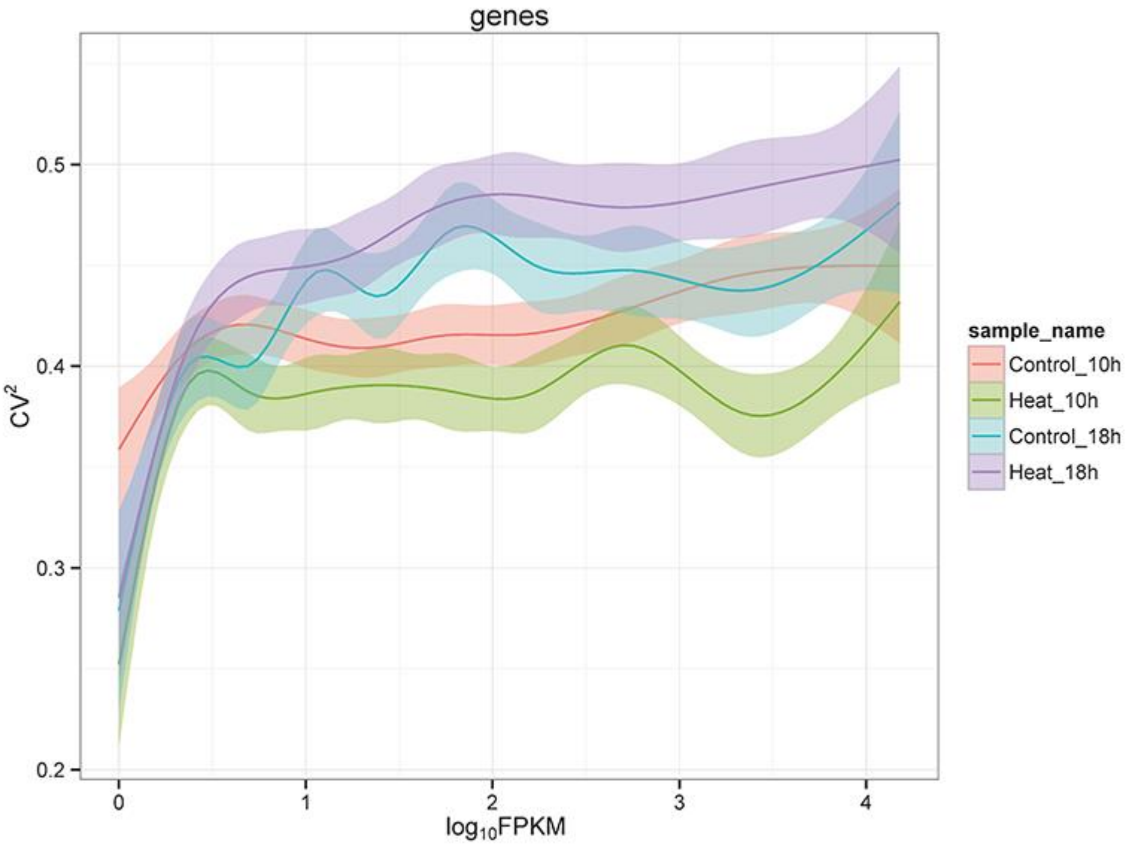

Supplementary Figure S2. The squared coefficient of the expression level of all genes in four transcriptome groups (Control_10h, Heat_10h, Control_18h and Heat_18h) of coral *G. fascicularis* after heat stress.

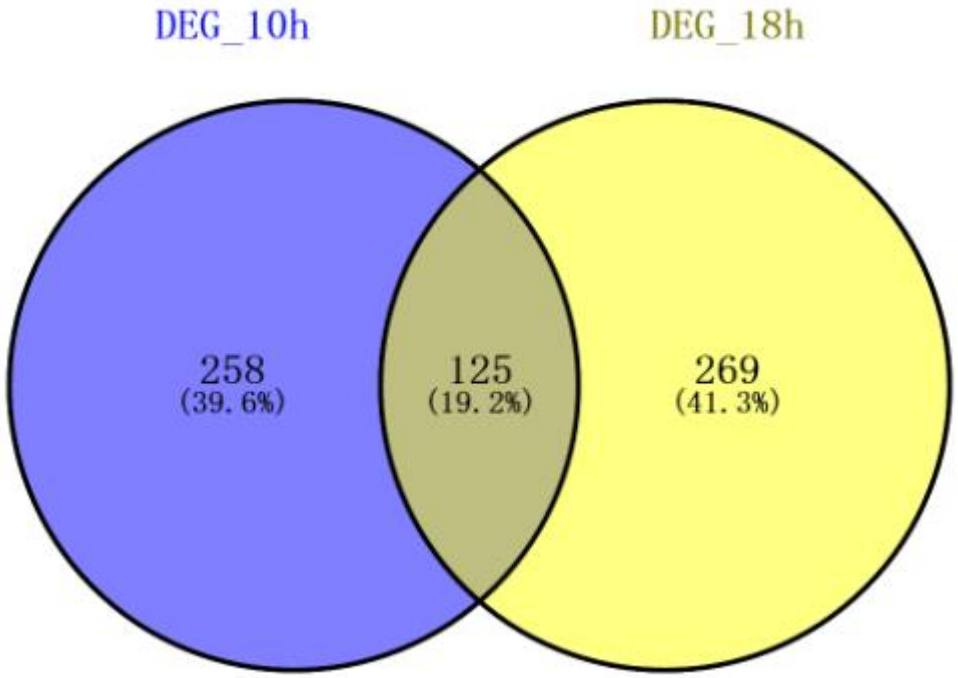

Supplementary Figure S3. Venn diagram showing the number of shared and unique significantly upregulated genes in comparisons Control_10h/Heat_10h (DEG_10h) and Control_18h/Heat_18h (DEG_18h).

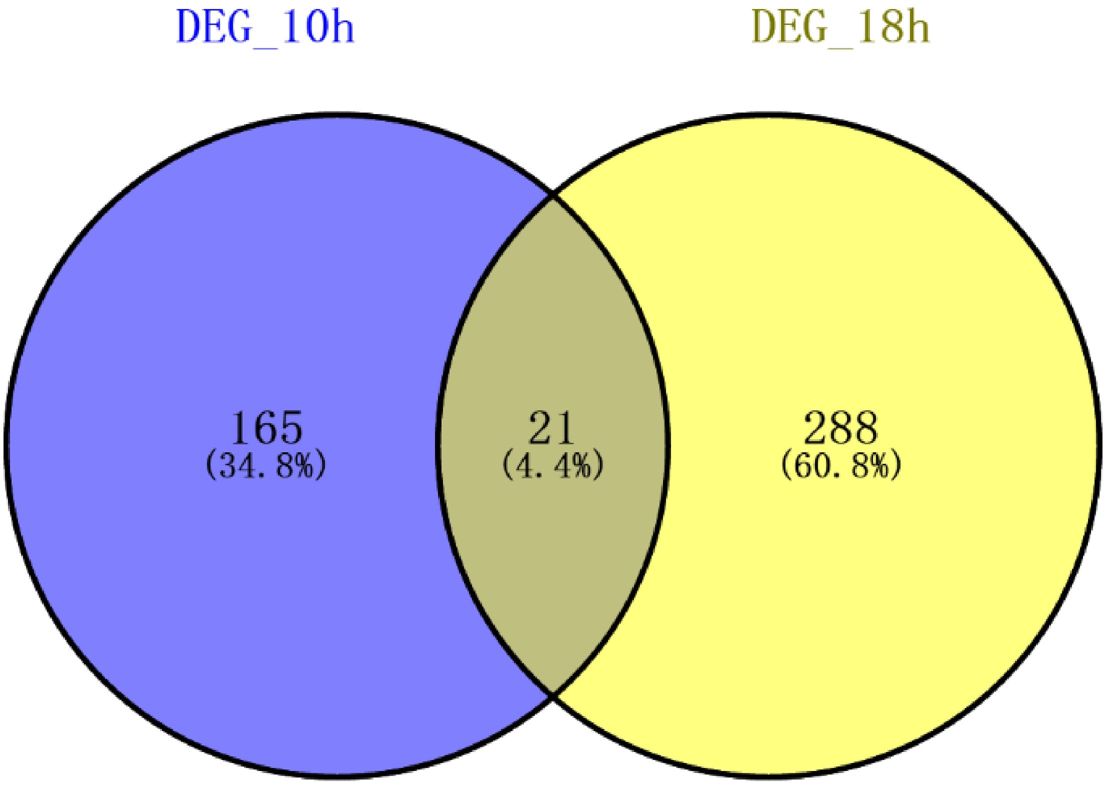

Supplementary Figure S4. Venn diagram showing the number of shared and unique significantly downregulated genes in comparisons Control_10h/Heat_10h (DEG_10h) and Control_18h/Heat_18h (DEG_18h).
